# The сontribution of FOXO family transcription factor genes (FOXO1, FOXO3) to chronic obstructive pulmonary disease

**DOI:** 10.18699/vjgb-25-90

**Published:** 2025-10

**Authors:** V.A. Markelov, L.Z. Akhmadishina, T.R. Nasibullin, Y.G. Aznabaeva, O.V. Kochetova, N.N. Khusnutdinova, S.M. Izmailova, N.Sh. Zagidullin, G.F. Korytina

**Affiliations:** Institute of Biochemistry and Genetics – Subdivision of the Ufa Federal Research Centre of the Russian Academy of Sciences, Ufa, Russia Bashkortostan State Medical University, Ufa, Russia; Institute of Biochemistry and Genetics – Subdivision of the Ufa Federal Research Centre of the Russian Academy of Sciences, Ufa, Russia Ufa State Petroleum Technological University, Ufa, Russia; Institute of Biochemistry and Genetics – Subdivision of the Ufa Federal Research Centre of the Russian Academy of Sciences, Ufa, Russia; Bashkortostan State Medical University, Ufa, Russia; Institute of Biochemistry and Genetics – Subdivision of the Ufa Federal Research Centre of the Russian Academy of Sciences, Ufa, Russia Bashkortostan State Medical University, Ufa, Russia; Institute of Biochemistry and Genetics – Subdivision of the Ufa Federal Research Centre of the Russian Academy of Sciences, Ufa, Russia; Bashkortostan State Medical University, Ufa, Russia; Bashkortostan State Medical University, Ufa, Russia; Institute of Biochemistry and Genetics – Subdivision of the Ufa Federal Research Centre of the Russian Academy of Sciences, Ufa, Russia Bashkortostan State Medical University, Ufa, Russia

**Keywords:** chronic obstructive pulmonary disease, cellular senescence, oxidative stress, FOX transcription factor family, FOXO1, FOXO3, хроническая обструктивная болезнь легких (ХОБЛ), клеточное старение, аутофагия, окислительный стресс, транскрипционные факторы семейства FOXO, FOXO1, FOXO3

## Abstract

Chronic obstructive pulmonary disease (COPD) is a multifactorial disease of the respiratory system and is the third leading cause of death worldwide. In the framework of the most relevant concepts of COPD pathogenesis, the key focus is on accelerated cellular senescence. FOXO family transcription factors are important hub components of cellular senescence signaling pathways. The objective of the study is to identify the association of FOXO1 (rs12585277, rs9549240), and FOXO3A (rs2253310, rs3800231) genes polymorphic variants with COPD and disease phenotypes. DNA samples from COPD patients (N = 710) and healthy individuals (N = 655) were used, polymorphic loci were analyzed by real-time PCR. For the first time, significant associations of FOXO1 (rs12585277) and FOXO3A (rs2253310) gene polymorphic loci with COPD and disease phenotypes were shown. Association with COPD was established with FOXO1 (rs12585277) (Padj = 0.0018, OR = 1.44 for the AG genotype) and FOXO3A (rs2253310) (Padj = 5.926 × 10–7, OR = 1.99 for the GG genotype). A significant genotype-dependent variation of smoking index (in pack/years), vital capacity and forced vital capacity was revealed for FOXO1 (rs9549240, rs12585277) and FOXO3A (rs2253310) loci. Multiple regression and ROC analysis identified highly informative COPD risk model, which included polymorphic variants of the FOXO1 (rs12585277) and FOXO3A (rs2253310) genes, smoking index and age (P = 5.25 × 10–93, AUC = 0.864). The multivariate regression model of the COPD “frequent exacerbator” phenotype included the AG genotype of FOXO1 (rs12585277), smoking index and age (AUC = 0.897, P = 4.1 × 10–86). FOXO family transcription factors involved in autophagy, oxidative stress and cellular homeostasis may provide a platform for a new diagnostic and treatment strategy for COPD as potential biomarkers and targets for therapy.

## Abstract (ru)

Хроническая обструктивная болезнь легких (ХОБЛ) – многофакторное заболевание дыхательной системы, является третьей ведущей (после ишемической болезни сердца и инсульта) причиной смерти в мире. В рамках наиболее актуальных концепций патогенеза ХОБЛ ключевое значение уделяется ускоренному клеточному старению. Транскрипционные факторы семейства FOXO – важные ключевые компоненты сигнальных путей клеточного старения. Цель исследования – выявление ассоциации полиморфных вариантов генов FOXO1 (rs12585277, rs9549240) и FOXO3A (rs 2253310, rs3800231) с развитием ХОБЛ и фенотипами заболевания. В работе использованы образцы ДНК больных ХОБЛ (N = 710) и здоровых индивидов (N = 655). Полиморфные локусы анализировали методом ПЦР в реальном времени. Впервые показаны значимые ассоциации полиморфных локусов генов FOXO1 (rs12585277) и FOXO3A (rs2253310) с ХОБЛ и фенотипами заболевания. Ассоциация с ХОБЛ установлена с локусами генов FOXO1 (rs12585277) (Padj = 0.0018, OR = 1.44 генотип AG) и FOXO3A (rs2253310) (Padj = 5.926 × 10–7, OR = 1.99 генотип GG). Обнаружена вариабельность показателей индекса курения (в пачках/ лет), жизненной емкости легких и форсированной жизненной емкости легких в зависимости от полиморф- ных вариантов локусов FOXO1 (rs9549240 и rs12585277) и FOXO3A (rs2253310). Идентифицирована значимая многофакторная регрессионная модель формирования ХОБЛ, в которую вошли полиморфные варианты генов FOXO1 (rs12585277) и FOXO3A (rs2253310), индекс курения и возраст обследуемых (P = 5.25 × 10–93, AUC = 0.864). Многофакторная регрессионная модель развития фенотипа ХОБЛ с частыми обострениями включала генотип AG локуса FOXO1 (rs12585277), индекс курения и возраст (AUC = 0.897, P = 4.1 × 10–86). Cвязанные с аутофагией, окислительным стрессом и клеточным гомеостазом транскрипционные факторы семейства FOXO как потенци- альные биомаркеры и мишени для терапии могут стать основой для разработки новой стратегии диагностики и лечения хронической обструктивной болезни легких

## Introduction

Chronic obstructive pulmonary disease (COPD) is a complex respiratory disease affecting the distal respiratory tract and pulmonary parenchyma with the development of pulmonary emphysema (Agustí et al., 2023). COPD is the third leading cause of death in the world, which explains the ongoing search for new approaches to diagnosis, treatment and prevention of the disease development (Agustí et al., 2023). Although intensive research has been conducted on both the molecular background and various clinical aspects of COPD, the mechanisms underlying the pathogenesis of COPD and different disease phenotypes remain incompletely understood (Brandsma et al., 2020).

COPD development results from exposure to a complex of risk factors over a long period of time, with tobacco smoking being the main one. Cigarette smoke exposure to airways leads to oxidative stress and activation of inflammatory cells and lung tissue damage (Domej et al., 2014). The most actual framework for COPD pathogenesis focuses on accelerated cellular senescence (Luo et al., 2024). As a fundamental mechanism for maintaining tissues and organs homeostasis, cellular senescence is being mediated by multiple processes. The most important of them include DNA damage, telomere loss, mitochondrial dysfunction, and autophagy and proteostasis alterations (Li et al., 2024). Previously, we have demonstrated the contribution of sirtuin family genes and the PI3K/AKT signaling cascade to COPD (Korytina et al., 2023). The FOXO transcription factors are major targets of the PI3K/AKT signaling cascade, exerting insulin-dependent regulation of cellular metabolism (Farhan et al., 2020). The mammalian FOXO class of transcription factors currently includes four proteins: FOXO1 (FKHR), FOXO3a (FKHRL1), FOXO4 (AFX), and FOXO6 (Santos et al., 2023). The FOXO transcription factors regulate the expression of a number of genes for antioxidant defense, cell cycle and apoptosis, proliferation, metabolism, and are involved in repression of mitochondrial respiratory chain proteins (Hagenbuchner et al., 2013).

The aim of this study was to identify the association of FOXO family transcription factor gene polymorphic variants (FOXO1, FOXO3) with COPD and disease phenotypes.

## Materials and methods

This study was designed according to the “case-control” principle. DNA samples from unrelated individuals, ethnic Tatars, who were residents of the Republic of Bashkortostan, were used in the study. The work was approved by the Ethics Committee of the Institute of Biochemistry and Genetics of the UFRC RAS (Protocol No. 19, dated November 1, 2022). Informed voluntary consent for the use of biological material in the study was obtained from all the participants. The inclusion and exclusion criteria had been described previously (Korytina et al., 2019). The diagnosis of COPD was established according to recommendations of the working group on the “Global Strategy for the Diagnosis, Treatment, and Prevention of Chronic Obstructive Pulmonary Disease” (http://goldcopd.org) on the basis of clinical and laboratory instrumental studies, including high-resolution computed tomography and spirometry.

The control group included unrelated individuals with no history of chronic diseases, including respiratory diseases and acute respiratory diseases at the time of biomaterial collection, matched for sex, age, smoking status, exposure to risk factors, region of residence, and ethnicity. The inclusion criteria in the control group were the normal parameters of respiratory function (FEV1/FVC > 70 %, FEV1 > 80 %) and age over 45 years. To identify genetic markers associated with COPD phenotypes, we performed a comparison between the control group and patients differentiated by disease phenotype as we described previously (Korytina et al., 2020) (Supplementary Table S1)1. Two phenotypes were distinguished: group 1 – COPD with frequent exacerbations; group 2 – patients with rare exacerbations. In the Table S1, the characteristics of the groups are summarized.


Supplementary Materials are available in the online version of the paper: https://vavilov.elpub.ru/jour/manager/files/Suppl_Markelov_Engl_29_6.pdf


Genotyping. DNA was isolated from peripheral blood leukocytes by phenol-chloroform extraction. The polymorphic loci of the FOXO1 (rs12585277, rs9549240) and FOXO3A (rs2253310, rs3800231) genes were selected; the functional significance of SNPs was analyzed by means of RegulomeDB Version 1.1 (https:// regulomedb.org), SNPinfo Web Server (https://snpinfo. niehs.nih.gov), and HaploReg v3 (Ward, Kellis, 2016) (Table S2). Bioinformatics analysis indicated that the selected SNPs affected gene function in different tissue types or were in linkage disequilibrium with functional loci of the gene. SNPs were analyzed using real-time polymerase chain reaction (PCR) with commercially available assays with fluorescent detection (https:// www.oligos.ru, DNA Synthesis LLC, Russia) using BioRad CFX96™ (Bio-Rad Laboratories, Inc, USA). The methods of analysis had been previously described by our group in detail (Korytina et al., 2019)

Statistical analyses. The description of standard statistical analysis methods was provided previously (Korytina et al., 2019). Analysis of deviation of obtained genotype frequencies from the Hardy–Weinberg equilibrium and the association of SNPs with the disease in the basic allele test and in regression models (dominant, recessive, additive (per rare allele dose – increase in the rare allele dose in the series: homozygote for frequent allele (0) – heterozygote (1) – homozygote for rare allele (2)) and overdominance (for heterozygotes)) were performed using the SNPassoc package v. 2.0–2 for R (González et al., 2007). An SNP was considered to be associated at P < 0.05; correction for multiple comparisons was conducted using the assessment method for the proportion of received false-positive results, false discovery rate (FDR), via online tool (https://tools.carbo cation.com/FDR). The haplotype frequencies and linkage disequilibrium structure LD (Dʹ, r2)) were calculated with Haploview 4.2. The contribution of polymorphic variants of the studied genes to the variability of quantitative traits indicating the severity of airway obstruction (lung function parameters – vital capacity (VC), forced vital capacity (FVC), forced expiratory volume in the first second (FEV1)) and smoking index was determined using linear regression. The logistic regression approach with stepwise imputation of independent variables was used to create multivariate regression models. The SNPs of the studied genes, as well as clinical and demographic parameters (age, smoking index, smoking status, sex) were selected as independent variables

A regression model is an equation in which the dependent variable is represented as a function of the independent variables (predictors). The log-regression equation has the form:

**form. 1.**
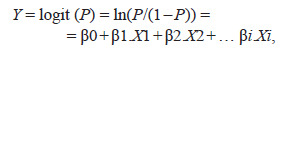
form. 1.

where Y is the dependent variable (status: 0 – control, 1 – case), X is the independent variable, β0 is a constant, β is the regression coefficient for the independent vari-able, X1...i is the value of the independent variable. The exponent of the individual regression coefficient β was interpreted as the odds ratio (OR). The hypothesis of significance of the whole model, taking into account all independent variables, was verified using the likelihood ratio (LR) test. To evaluate the effectiveness of the obtained prognostic model, the Nagelkerke R2 value and receiver operating characteristic (ROC) curve analysis were performed. The capacity of the regression model to discriminate between positive and negative cases (patient or healthy status) was assessed by the area under the ROC curve (AUC); the AUC value ranged from 0.5 (no discriminatory capacity of the model) to 1.0 (perfect discriminatory capacity). Calculations were carried out using SPSS v. 22.

## Results

The analysis of polymorphic loci of the FOXO1 (rs12585277, rs9549240) and FOXO3A (rs2253310, rs3800231) genes was carried out in the formed groups of COPD patients and the control group. The observed genotype frequencies of all examined SNPs in the control group were in accordance with the Hardy–Weinberg test: FOXO1 (rs12585277) (PH–W = 0.597), FOXO1 (rs9549240) (PH–W = 0.341), and FOXO3A (rs2253310) (PH–W = 0.3191), (rs3800231) (PH–W = 0.3831). Subsequently, we estimated the statistical significance of differences in distribution of allele and genotype frequencies between the groups, and the odds ratio values for the minor allele of each locus were calculated (basic allelic test) (Table 1).At the next stage, logistic regression was used to analyze the association of SNPs taking into account quantitative and binary traits (sex, age, smoking status and index), which were introduced into the regression equation as independent variables (Table 1).

**Table 1.**
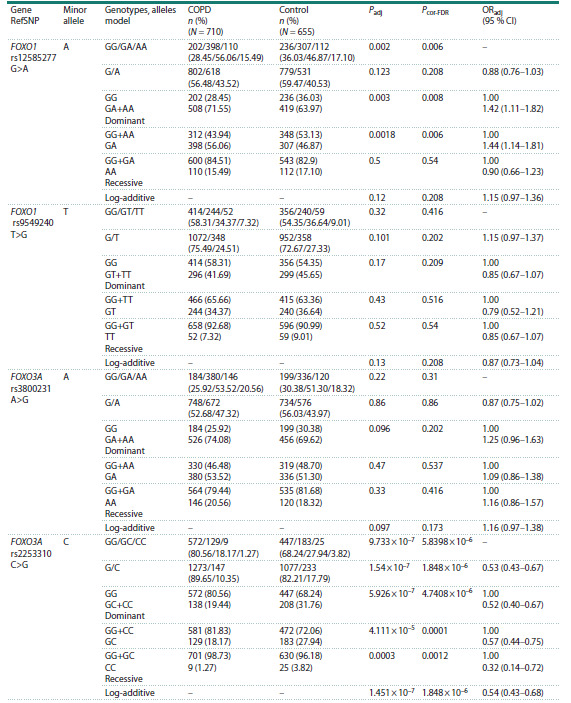
Allele and genotype frequencies of the examined polymorphic loci of the FOXO1 and FOXO3A genes in the groups of COPD patients and healthy individuals, association analysis with COPD Note. P – significance level of differences in allele and genotype frequencies between the groups (χ2 test for homogeneity of samples); regression analysis: number of individuals included in regression analysis (N = 1,365); Padj – significance of the likelihood ratio test of the log-regression model controlling for sex, age, and smoking status and index; ORadj – odds ratio controlling for all these factors, 95 % CI – 95 % confidence interval for OR; Pcor-FDR – test significance after correction.

Statistically significant differences in genotype frequency distributions between groups for FOXO1 (rs12585277) (P = 0.002) were found. The association with COPD was established in a dominant model (Padj = 0.003, OR = 1.42), the frequency of the heterozygous genotype in the COPD patient group reached 56.06 % versus 46.87 % in controls (Padj = 0.0018, OR = 1.44).

Significant differences in the distribution of genotype and allele frequencies between the patients group and healthy individuals were found for the FOXO3A (rs2253310) (P = 9.733 × 10–7 and P = 1.54 × 10–7, respectively). Association with COPD was found in dominant (Padj = 5.926 × 10–7, OR = 0.52), recessive (Padj = 3.360 × 10–3, OR = 0.32), log-additive models (Padj = 1.451 × 10–7, OR = 0.54), and with the heterozygous GC genotype (Padj = 4.111 × 10–5, OR = 0.57). Notably, the frequent G allele (P = 1.54 × 10–7, OR = 1.87 95 % CI 1.50–2.33) and the GG genotype (Padj = 5.926 × 10–7, OR = 1.99 95 % CI 1.57–2.47) were more often observed in the COPD group.

**Haplotype analysis of FOXO1 and FOXO3A genes polymorphic loci**

The linkage disequilibrium between loci rs12585277 and rs9549240 of the FOXO1 gene (Dʹ = 0.6183, r2 = 0.429) and statistically significant differences in the pattern of haplotype frequency distribution of the FOXO1 gene between the group of COPD patients and controls (P = 0.045) were revealed (Table S3). The frequency of the A-G haplotype at rs12585277 and rs9549240 loci was significantly higher in the group of COPD patients (24.26 vs. 18.81 % in controls, Padj = 0.011, OR = 1.33).

The linkage disequilibrium between polymorphic loci rs3800231 and rs2253310 of the FOXO3A gene was observed (Dʹ = 0.3315, r2 = 0.1452) (Table S3). Statistically significant differences in the haplotype frequency distribution pattern were established between the group of COPD patients and controls (P = 0.00001). The frequency of haplotype A-G at rs3800231 and rs2253310 loci was significantly higher in the COPD group (40.27 vs. 32.56 % in controls, Padj = 0.03, OR = 1.25), whereas haplotypes A-C and C-C were more frequent in the group of healthy individuals (Padj = 0.0076, OR = 0.65 and Padj = 0.0072, OR = 0.51, respectively).

**Association analysis of polymorphic variants of the FOXO1 and FOXO3A genes with different COPD phenotypes**

In order to identify genetic markers associated with COPD phenotypes, we performed a comparison of the control group and patients differentiated by disease phenotypes according to the GOLD classification (http:// goldcopd.org); this classification included an integral assessment of the COPD phenotype taking into account the number of exacerbations per year, results of COPD assessment test (CAT) and medical research council dyspnea scale (MRC), and lung function parameters (Table 2).

**Table 2.**
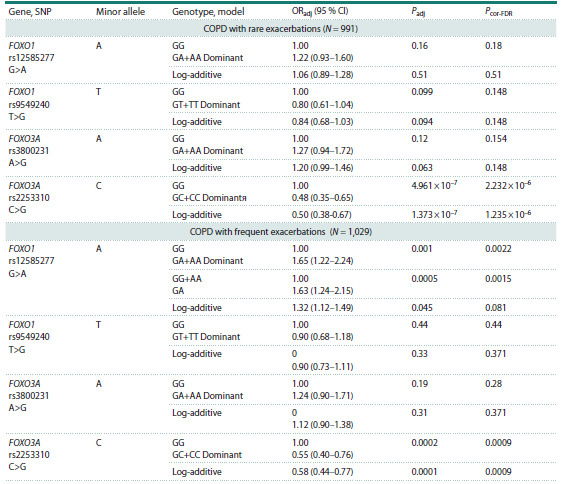
Results of association analysis of FOXO1 and FOXO3A gene polymorphic loci in groups differentiated by COPD phenotypes Note. N – number of individuals included in regression analysis; Padj – significance of the likelihood ratio test of the log-regression model controlling for sex, age, and smoking status and index; ORadj – odds ratio controlling for all these factors, 95 % CI – 95 % confidence interval for OR; Pcor-FD – test significance after correction

Statistically significant associations with the COPD phenotype with frequent exacerbations were obtained for FOXO1 (rs12585277) in the dominant model (Padj = 0.001, OR = 1.65) and for the heterozygous genotype (Padj = 0.0005, OR = 1.653); and for FOXO3A (rs2253310) in the dominant (Padj = 0.0002, OR = 0.55) and log-additive models (Padj = 0.0001, OR = 0.58). It should be noted that the homozygotes for the frequent allele G of FOXO3A (rs2253310) were more frequent in patients (OR = 1.81 95 % CI 1.32–2.49). The frequency of the A-G haplotype at rs12585277 and rs9549240 loci of the FOXO1 gene was higher in the COPD group (24.29 vs. 18.81 % in controls, Padj = 0.022, OR = 1.36 95 % CI 1.05–1.76) (Table S4).

In COPD patients with rare exacerbations, a significant association was confirmed only for FOXO3A (rs2253310) in the dominant (Padj = 0.00001, OR = 0.48) and log-additive (Padj = 0.00001, OR = 0.5) models. The GG genotype of FOXO3A (rs2253310) (OR = 2.09 95 % CI 1.53–2.88) and the A-G haplotype at rs3800231 and rs2253310 loci of FOXO3A (Padj = 0.032, OR = 1.31 95 % CI 1.02–1.67) were more frequent in the patient group.

**Association of FOXO1 and FOXO3A polymorphic loci with lung function parameters and smoking index**

Smoking is a major risk factor for COPD and a trigger for the oxidative stress that results in DNA damage and cell apoptosis (Domej et al., 2014). The analysis of the quantitative parameter indicating the smoking intensity and history (smoking index) in the smokers’ group, including both patients and healthy individuals, depending on the polymorphic variants of the FOXO1 and FOXO3A genes was performed (Table S5). It has been established that the GT genotype of FOXO1 (rs9549240) and the GG genotype of FOXO3A (rs2253310) are associated with higher smoking index values (P = 0.0042 and P = 0.012).

Lung function parameters including vital capacity (VC), forced vital capacity (FVC), forced expiratory volume in the first second (FEV1), ratio of forced expiratory volume in 1 s to vital capacity (FEV1/FVC) are key clinical variables indicating the degree of airway obstruction in COPD and disease progression. The indi viduals with the homozygous GG genotype of FOXO1 (rs9549240) had lower VC values (P = 0.0071); the carriers of the A allele in the homozygous and heterozygous state (dominant model) of FOXO1 (rs12585277) had lower FVC values (P = 0.04).

**Multiple regression and ROC analysis**

At the final stage, using multiple regression analysis with a stepwise forward inclusion of predictors followed by ROC analysis, a search for complex clinical-genetic models of COPD development was carried out. The genotypes or alleles of the studied genes were selected as independent variables, subsequently clinical and demographic variables (sex, age, smoking status and smoking index) were added and the most significant multivariate regression models were selected. The lung function parameters were excluded, as they are classical and well-identified predictors of COPD and disease severity.

The informative regression model of COPD development included polymorphic variants of FOXO1 (rs12585277) (AG genotype), and FOXO3A (rs2253310) (C allele) genes, smoking index and age of the subjects (P = 5.25 × 10–93) (Table 3). The ROC analysis of the model demonstrated its high ability to discriminate between COPD patients and healthy individuals (AUC = 0.864, sensitivity – 78.3 %, specificity – 82.3 %) (see the Figure a).

**Table 3.**
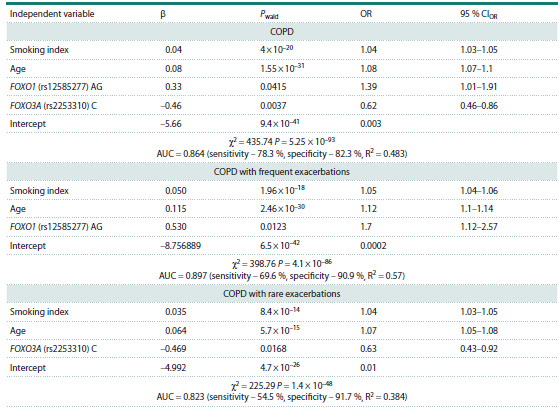
The predictive regression models for COPD development Note. β – the beta coefficient for the independent variable; Pwald – the significance for Wald statistics (Wald statistic is the regression coefficient divided by the square of the standard error: β/SE2), indicates the significance of the independent variable; OR – odds ratio, represents the exponent of the beta coefficient (expβ) for the independent variable; χ2 – the likelihood ratio test (LR), is necessary to test the hypothesis of the significance of the regression model, taking into account all independent variables; P – the value for the likelihood ratio test; R2 – Nagelkerke R2 model quality indicator – reflects the proportion of variability in the trait; intercept for the regression equation, the value of the dependent variable at which the independent variable is equal to zero; AUC – area under the curve; sensitivity – the proportion of correctly classified patients with a given diagnosis; specificity – the proportion of correctly classified healthy individuals. The ROC curves are presented in the Figure.

**Fig. 1.**
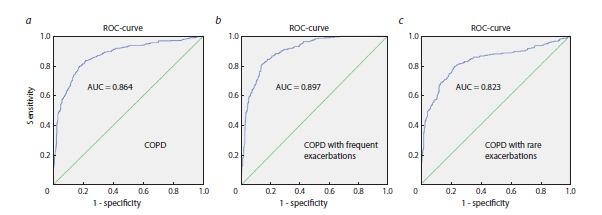
Area under the curve (ROC curve) to evaluate the effectiveness of prognostic regression models of COPD development (a); COPD phenotype with frequent exacerbations (b); COPD phenotype with rare exacerbations (c) including significant clinical, demographic and genetic predictors. AUC is the area under the curve. Full model characteristics are presented in Table 3.

A significant multifactorial regression model of COPD phenotype with frequent exacerbations included the AG genotype of FOXO1 (rs12585277), smoking index and age; the regression model was characterized by a high ability to discriminate between patients and healthy individuals (AUC = 0.897, P = 4.1 × 10–86). However, sensitivity, a measure of the model’s ability to correctly classify patients with a given COPD phenotype from healthy individuals, was only 69.6 %; at the same time, the model accurately classified healthy individuals, as it possessed high specificity (90.9 %) (see the Figure b).

The multifactorial regression model for COPD phenotype with rare exacerbations included the C allele of FOXO3A (rs2253310), smoking index and age (P = 1.4 × 10–48). The ROC analysis of the obtained model demonstrated its moderate discriminatory ability (AUC = 0.823), low sensitivity (54.5 %), but high specificity (91.7 %) (see the Figure c).

## Discussion

The association of FOXO1 (rs12585277) with COPD and the COPD phenotype with frequent exacerbations was established; haplotype A-G at rs12585277 and rs9549240 of FOXO1 was more frequent in the general COPD group and among patients with frequent exacerbations. The FOXO1 (rs12585277) AG genotype is part of multivariate regression model of COPD development and the COPD phenotype with frequent exacerbations, as well as predictors such as age and smoking index. A decline in lung function values indicating the severity of airway obstruction was revealed: FVC in carriers of homozygous and heterozygous genotype for the rare A allele of FOXO1 (rs12585277), and VC in carriers of the GG genotype of the FOXO1 locus (rs9549240). The variability of smoking index parameter was determined depending on FOXO1 (rs9549240) genotypes

FOXO1 is located on chromosome 13q14.11 (https:// www.ncbi.nlm.nih.gov/gene/2308). Previously, a number of polymorphic loci of FOXO1 have been shown to be associated with type 2 diabetes mellitus and obesity (Hussain et al., 2022; Santana et al., 2024). The studies on the association of polymorphic variants of the FOXO1 gene with COPD have not been conducted yet. T. Xue et al. (2024) have identified increased levels of FOXO1 mRNA and protein in the lung tissue of mice with lung emphysema model. Meanwhile, FOXO1 expression has been shown to be downregulated in the blood of COPD patients (Zhu et al., 2020).

The most significant associations with COPD were observed with FOXO3A (rs2253310); the G allele and the GG genotype were more frequent in the patient group. This association maintained significance regardless of the disease phenotype. The GG genotype was associated with an increased smoking index among all smokers; this quantitative measure characterizes smoking duration and intensity. The increase in smoking index is a major risk factor for COPD; the obtained results may be related to a higher prevalence of individuals with a high smoking index (more than 40 pack/years) among COPD patients with the GG genotype of FOXO3A (rs2253310). According to the multiple regression analysis results, the rs2253310 locus is a significant predictor for COPD development in general, in addition to FOXO1 (rs12585277), age and smoking index.

The FOXO3A gene is located on chromosome 6q21 (https://www.ncbi.nlm.nih.gov/gene/2309). The association of polymorphic variants in the FOXO3A gene with longevity (Soerensen et al., 2015) and a number of ageassociated diseases (Klinpudtan et al., 2022; Cao et al., 2023) has been shown. Association studies of FOXO3A gene polymorphic loci with COPD have not been previously conducted.

Systemic effects are typical for COPD, resulting in the development of severe complications that additionally exacerbate the disease progression in some patients (Agustí et al., 2023). Due to changes in the COPD diagnosis and prevention strategy (http://goldcopd.org), a great attention of researchers is currently focused on the identification of different disease phenotypes markers and effective determination of the patients with frequent exacerbations, as this category of COPD patients is characterized by a rapid progression of airway obstruction and increased mortality (Geerdink et al., 2016). It was shown that heterozygous genotype AG of FOXO1 (rs12585277) and genotype GG of FOXO3A (rs2253310) were significantly more frequent in the group of COPD patients with frequent exacerbations. The results of multivariate regression analysis demonstrated that the regression model for discriminating COPD patients with frequent exacerbations from healthy individuals had the highest efficiency parameters (such as AUC and R2 levels). It can be explained by the greater homogeneity of the patients’ group with this phenotype. The obtained data suggest that the most informative genetic marker for COPD phenotype with frequent exacerbations, among those that showed an association, is the AG genotype of FOXO1 (rs12585277).

The association of FOXO1 and FOXO3A gene polymorphic loci with COPD or disease phenotypes has not been studied before. For the first time, we investigated the role of genes encoding FOXO transcription factors in the disease development. The current interest is based on the fact that FOXOs regulate the expression of proteins related to autophagy, oxidative stress and cellular metabolism (Hagenbuchner, Ausserlechner, 2013; Gui, Burgering, 2022).

The activation of FOXO1 has been demonstrated to suppress oxidative stress-induced apoptosis of epithelial cells in a model of bronchopulmonary dysplasia (Zang et al., 2023). FOXO1 stimulates the expression ofa 150 kDa oxygen-regulated protein (ORP150) and in this way protects airway epithelial cells from endoplasmic reticulum stress mediated by cigarette smoke exposure (Liu et al., 2018). The inhibition of FOXO1 stimulates the related processes of autophagy and endoplasmic reticulum stress (Guo et al., 2022) and induces phenotypic conversion of pulmonary macrophages, which contributes to inflammation and airway remodeling (Chung et al., 2019).

It has been shown that FOXO3 activity suppresses cellular senescence and pathological airway remodeling induced by cigarette smoke exposure (Yao et al., 2012); on the other hand, inhibition of FOXO3 expression promotes the accumulation of NF-kB in the nucleus and stimulates its pro-inflammatory transcriptional activity (Di Vincenzo et al., 2018). All these processes are important pathogenetic mechanisms contributing to COPD development. The oxidative stress induced by cigarette smoke stimulates the transcriptional activity of FOXO3, leading to the activation of FOXO1 expression and stimulating its binding to the promoters of autophagy protein genes (ATG5, ATG12, ATG16), beclin protein 1 (BECLIN1) and microtubule-associated protein 3 alpha light chain 1 (LC3) genes (Bagam et al., 2021). The antioxidant function of FOXO3 is determined by increased expression of the SOD2, CAT, and GPX1 genes, which play a key role in the regulation of reactive oxygen species (ROS) homeostasis in lung cells as a response to oxidative stress (Mahlooji et al., 2022).

H. Jiang et al. (2023) established that activation of FOXO3A under stress factors leads to cellular adaptation and reduced cellular senescence, whereas suppression of FOXO3A activity is associated with greater mitochondrial injury in pulmonary epithelial cells. The insufficient level of FOXO3, which may be associated with functional polymorphic variants as well, leads to suppression of antioxidant gene expression, resulting in the development of oxidative stress as a response to cigarette smoke exposure (Hwang et al., 2011).

Therefore, FOXO transcription factors play a key role in the normal functioning of mitochondria, preventing the oxidative stress and in this way inhibiting the progression of lung epithelial cellular senescence (Chen et al., 2021), a critical pathogenetic mechanism of COPD development.

## Conclusion

For the first time, significant associations of FOXO1 (rs12585277) and FOXO3A (rs2253310) gene polymorphic loci with COPD and disease phenotypes have been shown. The FOXO family transcription factors related to autophagy, oxidative stress and cellular homeostasis, as potential biomarkers and targets for therapy, may provide a platform for a new diagnostic and treatment strategy for COPD.

## Conflict of interest

The authors declare no conflict of interest.
